# RNA-Binding Proteins: Modulators of Canonical Wnt Signaling Pathway

**DOI:** 10.3390/ijms27010205

**Published:** 2025-12-24

**Authors:** Michael S. Czap, Vikash Singh, Vladimir S. Spiegelman

**Affiliations:** Division of Pediatric Hematology and Oncology, Department of Pediatrics, The Pennsylvania State University College of Medicine, Hershey, PA 17033, USA; mczap@pennstatehealth.psu.edu (M.S.C.); vsingh2@pennstatehealth.psu.edu (V.S.)

**Keywords:** RBP (RNA binding protein), Wnt/β-catenin, untranslated region (UTR), N^6^-Methyladenosine (m^6^A), RNA recognition motif (RRM), heterogenous nuclear K-homology (KH)

## Abstract

RNA-binding proteins (RBPs) play a pivotal role in post-transcriptional gene regulation, influencing various cellular processes, including development, differentiation, and disease progression. Emerging evidence suggests that RBPs function as critical modulators of the canonical Wnt signaling pathway, a key regulator of cell fate determination, proliferation, and tumorigenesis. By controlling the stability, localization, and translation of Wnt pathway components, RBPs fine-tune the dynamic signaling responses necessary for maintaining cellular homeostasis. Several RBPs have been identified as direct regulators of key components in the Wnt cascade, such as IGF2BP1, HuR, and MSI1, impacting their expression and activity. Dysregulation of these RBPs has been linked to aberrant Wnt signaling, contributing to various pathological conditions such as cancers or developmental disorders. This review explores the emerging landscape of RBPs in the regulation of canonical Wnt signaling, highlighting their molecular mechanism, functional implications, and potential as therapeutic targets in Wnt-driven disease.

## 1. Introduction

Precise control of cell growth and proliferation is fundamental to tissue homeostasis, and disruption of these regulatory mechanisms causes many pathologies, including cancers. Wnt signaling is one of the key pathways that orchestrates development, stem cell maintenance, and tissue regeneration. Aberrant Wnt activity, whether through genetic, epigenetic, or environmental perturbations, can drive pathological proliferation, impaired differentiation, and tumorigenesis, highlighting its critical role in both physiological regulation and disease.

The Wnt family of protein ligands, generally, are growth factors that can stimulate proliferation, direct embryogenesis, and prevent differentiation in adult stem cells [1,2,3]. The highly conserved canonical Wnt/β-catenin signaling pathway includes the Wnt1 class of Wnt proteins (Wnt2, Wnt3, Wnt3a, and Wnt8a; Wnt3a being the primary extracellular ligand), and triggers a signaling cascade that results in the transcription of specific Wnt/β-catenin target genes [4].

On the surface of the cell, the seven-pass transmembrane protein Frizzled (FZD) remains separated from low-density lipoprotein receptor-related proteins 5 and 6 (LRP5/6). This separation is a result of the extracellular association of LRP5/6 with Dickkopf-1 (DKK-1), which inhibits the LRP5/6 interaction with FZD by inducing its endocytosis [5,6]. Concurrently, β-catenin is sequestered to the Destruction Complex (DC) (comprising casein kinase 1 (CK1) and glycogen synthase kinase 3 (GSK3β), APC, βTrCP, and scaffolding protein Axin) as well as by E-cadherin, reinforcing its deactivation [7]. When Wnt interacts with FZD and LRP5/6 on the surface, the intracellular domain of FZD undergoes a conformational change and recruits Disheveled (Dvl) to the intramembrane, thus allowing association with the intracellular domain of LRP5/6 and recruitment of Axin, CK1, and GSK3β [8,9]. RNF43/ZNRF3 are transmembrane E3 ubiquitin ligases that reduce Wnt receptor (FZD and LRP5/6) levels on the cell surface by promoting their degradation, while R-spondin prevents this by binding to RNF43/ZNRF3, resulting in increased receptor availability and enhanced Wnt signaling [10]. The recruitment of these core DC proteins away from β-catenin causes the complex to collapse and β-catenin to accumulate in the cytoplasm. β-catenin can then translocate into the nucleus, where it associates with T-cell factor/lymphoid enhancer factor (TCF/LEF) to bind to specific promoter regions of DNA, activating the transcription of targeted genes [3,11,12]. Some transcriptional targets of β-catenin/TCF/LEF include genes that promote proliferation, such as Cyclin-D1, c-Myc, Axin-2, and DKK-1 [6,13,14,15]. Notably, the transcriptional activation of Axin-2 and DKK-1 represents negative feedback loops induced by β-catenin/TCF/LEF upregulation and is an example of β-catenin-mediated autoregulation.

RNA-binding proteins (RBPs) have emerged over the last several decades as important post-transcriptional regulators of gene expression. 838 out of about 3030 identified human RBPs have been shown to be associated with diseases [16]. The presence of hallmark domains that are known to interact with RNA (such as the KH, RRM, and zinc finger families) allows these proteins to fall into the category of canonical RBPs. The RNA-binding domains of such proteins use a variety of interactions to recognize and bind their target RNAs, with each domain displaying unique binding mechanisms and specificity [17]. Although a single domain’s interaction can be complex and multi-faceted, the binding affinity and specificity are usually enhanced through the modular combination of several domains, their organization, and the properties of linker regions, allowing recognition of different RNA sequences and structures as well as providing regulatory flexibility [18]. The RNA–protein interactions thus form ribonucleoprotein (RNP) complexes, which regulate the post-transcriptional processes necessary for gene regulation. The RNPs are involved in every level of mRNA fate determination, including metabolism, splicing, processing, localization, stability, and translation [19]. Many RBPs are reported to regulate the canonical Wnt signaling pathway. Dysregulation of these RBPs can alter Wnt signaling and contribute to the progression of diseases such as cancers and confer resistance to therapy. Understanding this relationship offers promising avenues for developing targeted cancer treatments by modulating RBPs to affect Wnt signaling and overcome drug resistance, thereby improving patient outcomes. Although RBP-mediated regulation of Wnt signaling is diverse and context dependent, we focus here on the most relevant examples that illustrate key regulatory and autoregulatory mechanisms with the greatest potential for therapeutic translation in cancer (Table 1).

## 2. RBPs in Regulation of Canonical Wnt Signaling

### 2.1. IGF2BP1

The IGF2BP family of proteins, comprising IGF2BP1, IGF2BP2, and IGF2BP3, represents a group of RBPs that play essential roles in post-transcriptional gene regulation. These proteins contain six conserved RNA-binding domains, two RRMs at the N-terminus and four heterogeneous nuclear K-homology (KH) domains at the C-terminus. Working cooperatively, these domains recognize and bind specific RNA motifs, particularly GG(m^6^A)C sequences [37]. KH domains are essential for RNA binding, the trafficking of target mRNA, and the formation of RNP granules [38]. The KH1/2 domains were shown to stabilize IGF2BP-RNA complexes, while the KH3/4 domains are critical for recognizing and binding m^6^A-modified target RNAs [39]. The IGF2BP family of proteins regulates RNA stability in cancer through multiple mechanisms, such as protecting mRNAs from RISC and miRNA-mediated degradation, as well as acting as m^6^A readers to stabilize specific transcripts [40].

Among this family, IGF2BP1 (also known as IMP1/CRD-BP/ZBP1/VICKZ1) has attracted particular interest because of its prominent role in canonical Wnt/β-catenin signaling [20]. IGF2BP1 is both a direct transcriptional target of β-catenin/TCF complexes and a post-transcriptional regulator of Wnt pathway components [41]. IGF2BP1 expression is strongly induced by Wnt signaling and, in turn, stabilizes a wide range of oncogenic mRNAs, including c-MYC, Wnt ligands and receptors, and Wnt signaling components such as βTrCP1 and GSK-3β [20,21,42,43,44,45,46]. In one of its earliest reports, IGF2BP1 was shown to stabilize *βTrCP1* mRNAs in response to β-catenin signaling, thereby facilitating a critical negative feedback loop of Wnt signaling that exists only in cells permitting the intact phosphorylation of β-catenin (i.e., normal cells that lack mutations in APC or β-catenin) [20]. In many cancers, IGF2BP1 amplifies Wnt/β-catenin pathway output through feed-forward regulatory mechanisms that influence downstream effectors such as *c-MYC* [20].

**Figure 1:**
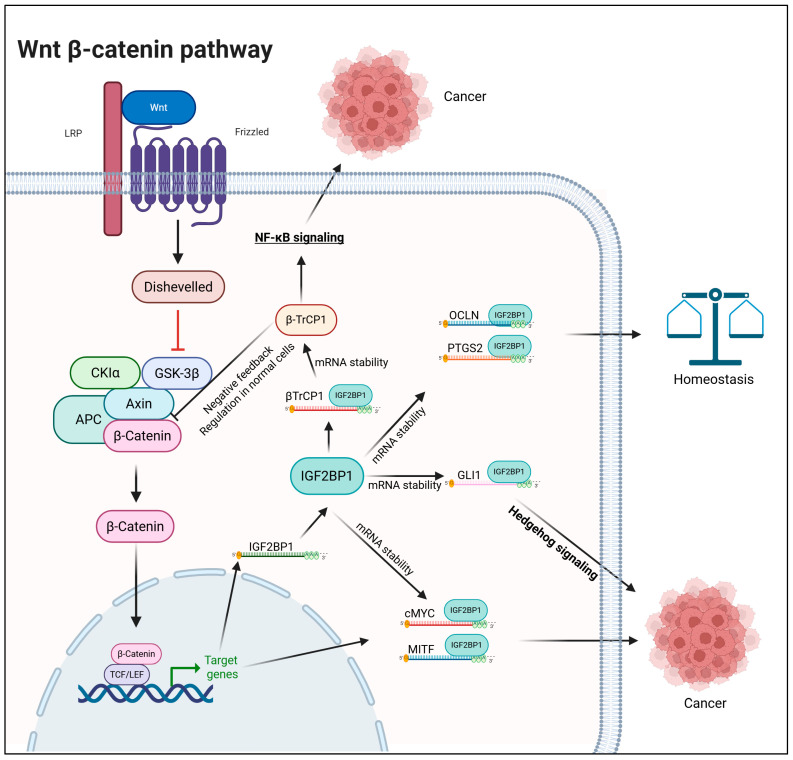
Regulation of Wnt signaling by IGF2BP1. Created in BioRender. Singh, V. (2025) https://BioRender.com/5rpbn1h (accessed on 24 November 2025).

At a transcriptome-wide level, IGF2BP1 emerges as a key regulator of Wnt-driven gene expression. In non-transformed cells, about one-third of Wnt-induced transcripts show IGF2BP1 dependency, with some, such as PTGS2 and OCLN, being direct RNA-binding targets [43,47]. In CRC cells with constitutively active Wnt signaling, reliance on IGF2BP1 is substantially greater, with nearly three-quarters of genes suppressed by β-catenin/TCF7L2 inhibition being restored upon IGF2BP1 overexpression. Integrated RNA-seq and TCF7L2 ChIP analyses further identify two classes of genes: (i) those directly regulated by Wnt/β-catenin and further stabilized by IGF2BP1, and (ii) those influenced by Wnt signaling only indirectly—through IGF2BP1-mediated post-transcriptional control [41]. IGF2BP1-mediated crosstalk between Wnt signaling and other pathways has also been reported. Wnt signaling has been shown to potentiate NF-κB activity through IGF2BP1-mediated stabilization of *βTrCP1* mRNA [20]. Another study demonstrated that Wnt signaling enhances Hedgehog pathway activation by stabilizing *GLI1* mRNA, thereby amplifying its transcriptional output (Figure 1) [44].

Additional studies extend these findings to other cancers. In cervical cancer, exosome-derived miR-1323 from cancer-associated fibroblasts downregulates PABPN1, which then recruits IGF2BP1 to stabilize GSK-3β mRNA, activating Wnt/β-catenin signaling and promoting tumor progression and radio resistance [21]. In osteosarcoma, the m^6^A eraser FTO reduces *DACT1* mRNA stability, correlating with poor survival. IGF2BP1 counteracts this effect by stabilizing DACT1 transcripts, thereby sustaining Wnt/β-catenin activity and influencing disease progression [48]. Interestingly, a positive feedback loop of regulation was seen in the case of β-catenin and c-MYC, where in lung adenocarcinoma, the circular RNA circXPO1 binds IGF2BP1 and enhances the stability of β-catenin mRNA, driving Wnt/β-catenin activation, tumor growth, and poor clinical outcomes [49]. Likewise, in a separate finding, c-MYC was shown to regulate the transcriptional activity of IGF2BP1 [45]. Together, these findings identify IGF2BP1 as both an amplifier and a reprogrammer of transcriptomes in Wnt-driven normal and cancer cells.

Given the fact that IGF2BP1 was shown to play an important role in the progression of a variety of malignancies, it has become an attractive therapeutic target, prompting the development of specific IGF2BP1 inhibitors such as BTYNB, 7773, AVJ16, and A11 [50,51,52,53,54,55]. BTYNB has been shown to reduce cellular levels of *cMYC* mRNA and protein in melanoma and ovarian cancer, while AVJ16 was shown to reduce the expression of *BTRC* mRNA [51,55]. These data suggest that pharmacological inhibition of IGF2BP1 function can significantly affect Wnt signaling outcomes in cancer. Studies focusing on the synergistic effects of combining Wnt inhibitors that are currently in clinical trials with IGF2BP1 inhibitors represent an important area of ongoing and future research.

### 2.2. ELAVL1 (HuR)

A member of the ELAV-family of RNA-binding proteins [56], ELAV-like protein 1 (ELAVL1), also referred to as Hu antigen R (HuR), is one of the best-studied post-transcriptional mRNA fate determinants [57,58,59]. Functionally, HuR shuttles between the nucleus and cytoplasm to stabilize, localize, and promote translation of mature transcripts [60,61,62]. These roles underscore the oncogenic potential of HuR in multiple types of cancer, including breast, colon, lung, and others [58]. Like the other members of the Hu family, HuR contains three conserved RRM domains with RRM2 and RRM3 being separated by a variable hinge region [63]. Within the hinge region resides the HuR nucleocytoplasmic shuttling sequence (HNS) that allows HuR to shuttle between the nucleus and cytoplasm [60,64]. HuR export depends on CRM1 (nuclear export protein)-mediated recognition of its leucine-rich nuclear export signal (NES), while its import into the nucleus occurs via a classical nuclear localization signal (NLS) complex formed with Transportin 2 (TRN2). This process is driven by nuclear RanGTP levels that cause dissociation of the HuR-TRN2 complex and allow TRN2 to recycle to the cytosol and import new HuR molecules from the cytoplasm [65,66,67,68]. Dysregulation of these processes has been shown to result in enhanced tumorigenesis and metastasis as well as worse patient prognosis [59,69].

Mounting evidence continues to demonstrate the dependency of the Wnt signaling cascade on HuR regulation. Of the >4000 HuR mRNA targets [70,71], two have been found within the canonical Wnt pathway. Within the intestinal epithelium, HuR binds to the 3′-UTR of LRP6, stabilizing it and enhancing translation. This enhancement of LRP6 is required for normal small intestine mucosal growth [22]. A similar mechanism was also observed with HuR overexpression in an ovariectomized experimental mouse model, which helped counteract osteoporosis-related effects [72]. These reports indicate that HuR plays an essential role in maintaining normal tissue and organ homeostasis by regulating Wnt signaling.

**Figure 2:**
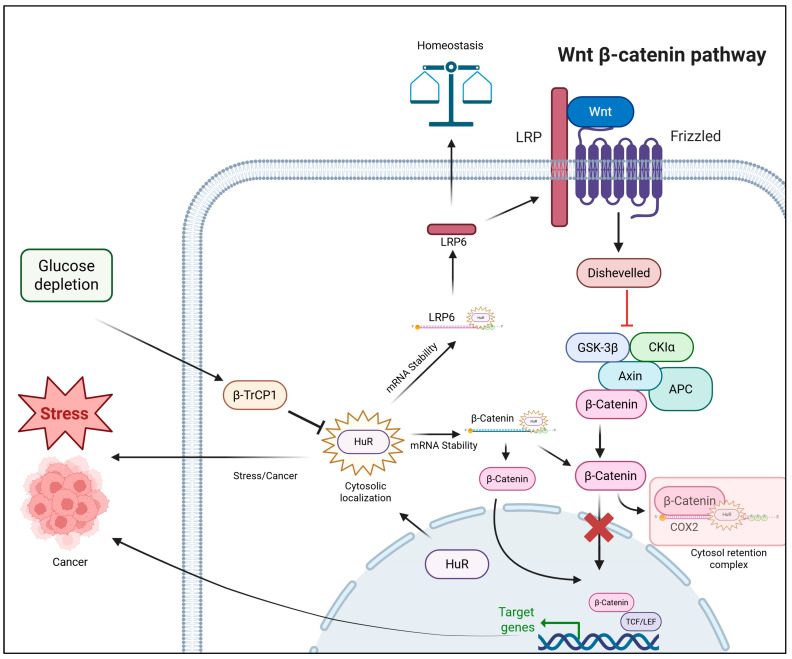
Wnt modulation by HuR/ELAVL1. Created in BioRender. Singh, V. (2025) https://BioRender.com/4687r1w (accessed on 24 November 2025).

HuR was also reported to be a direct regulator of *CTNNB1* mRNA. Early evidence demonstrated that HuR expression increases in parallel with colon cancer progression [73]. Subsequent work in rat liver epithelial cells showed a positive regulatory relationship between β-catenin and HuR levels [74]. In one study, it was reported that HSF1 upregulates HuR, which in turn stabilizes and enhances β-catenin mRNA translation, leading to increased β-catenin levels that reinforce its function as a key oncogenic driver in breast cancer [75]. Transcriptomic analyses suggest CTNNB1 to be a binding target of HuR, as well as its ability to stabilize *CTNNB1* mRNA [23,70,71]. Long noncoding RNA TSLNC8 associates with HuR protein and promotes its interaction with and increases stabilization of *CTNNB1* mRNA [76]. In colon cancer cells, β-catenin recruits HuR to *COX-2* mRNA in the nucleus, stabilizing the transcript and promoting the formation of an RNP complex that translocates from the nucleus to the cytosol, which in turn diminishes β-catenin’s transcriptional activity [77,78,79]. In another study, a Wnt signaling component, βTrCP1, directs HuR degradation when glucose metabolism is inhibited, highlighting a potential cancer therapeutic strategy (Figure 2) [80].

The direct role of HuR-dependent stabilization of *CTNNB1* mRNA could prove to be a powerful tool for understanding many cancers with overlapping HuR expression and Wnt signaling activation. With the ongoing development of various HuR inhibitors (such as MS-444 and CMLD2), it is possible to modulate Wnt signaling in multiple different cancers, suggesting these inhibitors may serve as potential therapeutic agents [50]. While current knowledge is limited, the significant interest in HuR shows promise to better establish its role in canonical Wnt signaling.

### 2.3. Musashi

Aside from its fame as a Japanese samurai, Musashi1 (MSI1) is most well-known for its effects on signal transduction pathways through translation regulation. Named for the sensillum phenotype of *Drosophila*, reminiscent of the ‘dual swords’ used by historical samurai, MSI1 was initially found to be essential for neuronal progenitor fate determination [81,82]. Since then, MSI1’s functions have expanded to a key role in mammalian multipotent stem cell population maintenance in neural, breast, colon, and bone marrow tissues, as well as in cancers [83].

Members of the highly conserved Musashi family, MSI1 and MSI2, contain two N-terminal, tandemly arranged RRM domains (RRM1 and RRM2), which facilitate their RNA-binding ability [81,83,84,85]. Within each of the MSI1 RRMs resides an NLS followed by a C-terminal NES, facilitating nuclear shuttling [86,87]. G/AU_1–3_AGU consensus sequence binding of RRM1 accounts for the majority of MSI1 RNA-binding activity, while RRM2 stabilizes the formation of RNA–protein complexes by recognition of UAG sequences [88,89,90]. The trans-acting MSI1 regulates translation post-transcriptionally through the binding of hairpin-like loops within target transcript 3′UTRs, allowing C-terminal interaction with PABP and inhibiting 80S ribosome formation [88,90,91,92,93]. MSI1 indirectly modulates mRNA stability via miRNA processing through C-terminal binding of Lin28, allowing its nuclear localization and inhibiting let-7 miRNA biogenesis [86]. MSI1 holds significant prognostic value for its upregulation in a variety of human malignancies.

**Figure 3:**
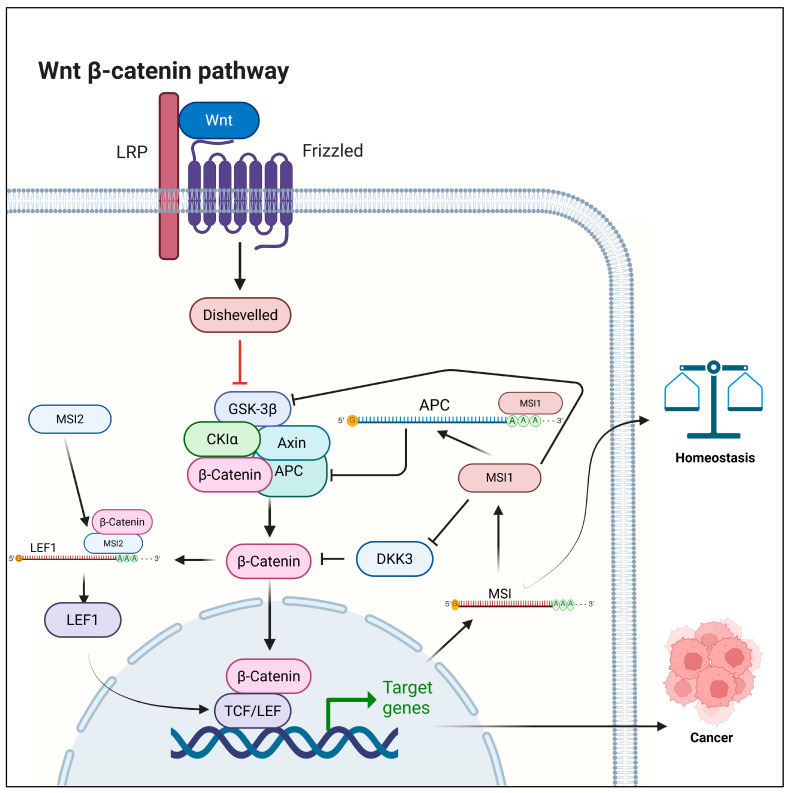
Wnt signaling modulation by MSI1 and MSI2. Created in BioRender. Singh, V. (2025) https://BioRender.com/i60e4ca (accessed on 24 November 2025).

MSI1 plays multiple context-dependent roles regulating canonical Wnt. A positive-feedback-loop mechanism between MSI1 and Wnt signaling was first identified in intestinal progenitor cells, in which MSI1 expression was induced by TCF4 binding to its promoter, while MSI1 mediated the expression of Frat1, a potent Wnt activator [94]. In a separate study, TCF4 binding to the promoter of MSI1 and the resulting increase in its expression were demonstrated in mouse intestinal crypts in response to ionizing radiation, suggesting that MSI1 is a target of Wnt/β-catenin signaling [95]. Additionally, the direct role of MSI1 was found by Spears and Neufeld, in which they observed MSI1 binding to the 3′UTR of *APC* mRNA, repressing its translation and its own transcription in a double-negative feedback loop in colon epithelia [24]. Interestingly, a later study utilizing HEK293 cells confirmed this MSI1-*APC* mRNA binding as well as identified MSI1 binding to the 3′UTR of CTNNB1, but failed to observe MSI1-Wnt potentiation in mouse MSI1-overexpressed epithelia models [25]. An indirect role of MSI1 was found in its downregulation of DKK3 expression as well as its secretion of DKK3 inhibitor PLF1 in mammary progenitor cells [96]. These results establish MSI1 as a regulator of Wnt signaling that maintains homeostasis in the intestine and mammary tissues (Figure 3).

MSI1 was also found to positively correlate with Wnt activation in hepatocellular carcinoma (HCC), and this correlation was shown in patients with poor prognosis [97,98]. Another indirect role of MSI1 was established in modulating Wnt signaling, where silencing MSI1 led to the downregulation of EMT markers and inhibition of Wnt pathway activities in cervical cancer (Figure 3) [99].

MSI2 is essential for WNT/β-catenin signaling in hematopoietic stem cells (HSCs), where it interacts with β-catenin and regulates the expression and nuclear localization of LEF-1 post-transcriptionally by binding to mRNA of *LEF1*, a key Wnt transcription factor. This interaction influences Wnt pathway activity and supports HSC proliferation [26]. MSI2 exhibits a similar regulatory effect on Wnt signaling in both myeloid leukemia cell lines and primary AML specimens (Figure 3) [100].

Stem cell population maintenance is a critical function within epithelial tissues throughout the body and in cancers, and is closely tied to the proper firing of the Wnt cascade. Given the complexity of MSI’s role in regulating Wnt signaling, these recent advancements point to potential for therapeutic development in epithelial and other tissues, highlighting the necessity for further study.

### 2.4. RBM Family

The RNA-Binding Motif (RBM) family of RBPs is an extensive and well-documented RBP family with functions at several levels of post-transcriptional regulation, including RNA splicing, metabolism, stability, localization, and translation [101,102]. While ‘RNA-Binding Motif’ refers to the family of RBM proteins, it also refers to the distinct structural motif within the RBM family. Components of these 90 amino acid motifs, RNA recognition motifs (RRM), are conserved across species, and are made of two consensus ribonucleoprotein (RNP) sequences, RNP-1 and RNP-2, which are separated by roughly 25–35 amino acids [103,104,105,106].

Several RBM family members have critical roles in tumorigenesis and disease development, exhibiting oncofetal patterns of expression. RBM3 is an essential protein for cell proliferation and has functions in protection against environmental stressors such as hypo- and hyperthermia, serum depletion, radiation, and toxins [107,108,109]. The ability of RBM3 to stabilize and promote the translation of mRNAs is also critical to its role in protecting mitosis under stress [110]. RBM3 contains an N-terminal RRM and a C-terminal arginine-glycine-rich (RGG) domain [109,111,112]. The former is primarily involved in 5′ capping and splicing, while the latter is involved in mRNA export and nuclear shuttling [113,114,115,116]. Considered as a proto-oncogene, RBM3 dysregulation is found in a variety of cancers, including CRC, esophageal, urethral, prostate, melanoma, and others [109]. Its family member, RBM47, originally identified for its importance in vertebrate embryo viability and growth, is a crucial regulator of RNA C-to-U editing, splicing, and stability [117,118,119,120,121]. Structurally, RBM47 contains three N-terminal RRMs and a C-terminal arginine-rich region, with the RRMs facilitating RNA binding featuring a preference for 3′UTR AREs [28,117,121,122]. RBM47 also contributes to the tumorigenesis of several cancers, including nasopharyngeal, breast, CRC, and lung [101].

**Figure 4:**
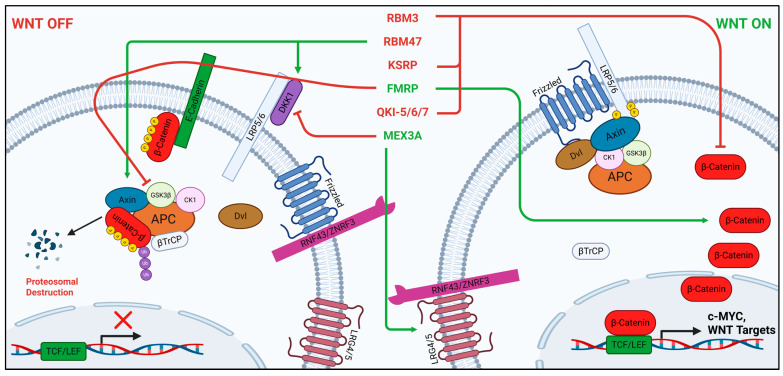
Modulation of canonical Wnt signaling mRNA by RNA-binding proteins RBM3, RBM47, KSRP, FMRP, QKI, and MEX3A. Created in BioRender. Czap, M. (2025) https://BioRender.com/pybj7j8 (accessed on 24 November 2025).

Both of these RBM family members have roles in the direct regulation of Wnt signaling. In a recent study, RBM3 has been found to bind directly to the 3′UTR of *CTNNB1* mRNA, enhance its METTL3-mediated m^6^A modification, and decrease its stability in prostate cancer cells cultured under a bone metastasis-mimicking microenvironment [27]. Contrary to this finding, a previous study identifies enhanced nuclear localization of β-catenin in response to RBM3 overexpression in CRC cells [123]. These studies highlight the context-dependent effects of RBM3 in cancer, demonstrating the requirement for new research in clinical applications. Additionally, RBM47 has been found to play multiple roles with respect to Wnt signaling. RBM47 has been found to bind to the 3′UTR of *DKK1* mRNA and stabilize it, resulting in tumor suppression via Wnt inhibition in breast cancer [28]. Likewise, RBM47 was later found to bind to the 3′UTR of *AXIN1* mRNA and stabilize it, leading to the disruption of Wnt/β-catenin signaling and tumor progression in NSCLC (Figure 4) [29]. These results agree with previous studies showing that the knockdown of RBM47 in zebrafish also leads to an increase in Wnt8a signaling [117], as well as increased CRC tumor burden and lower survival in *Apc^Min/+^* mice [124].

Another RBM family protein, RBM10, plays an indirect role in Wnt signaling via its RNA-binding capacity. In 2022, it was shown that RBM10 directly binds with the β-catenin suppressor CTNNBIP1 protein in LUAD, and further defines its previously less-understood tumor suppressor role by negatively regulating Wnt/β-catenin signaling through disruption of the β-catenin/TCF4 interaction [125,126,127]. RBM family member RBMS3 has recently been shown to bind to the 3′UTR and repress expression of TWIST1 in breast cancer, inhibiting migration and invasion [128]. In turn, TWIST1 has been found to indirectly regulate Wnt by upregulating AXIN2 in ovarian cancer [129].

Though there are only a handful of studies demonstrating the RBM family’s effects on canonical Wnt/β-catenin activity, it is clear that many within the RBM family of proteins share regulatory roles in Wnt activation. Future work is necessary to elucidate viable treatment options in a variety of cancers, as well as identify other potential RBM Wnt effectors.

### 2.5. KSRP

The multifaceted hnRNP (KH)-type splicing regulatory protein (KSRP or KHSRP) contributes to gene expression at various levels, including transcription, mRNA splicing, mRNA maturation, mRNA localization, and mRNA decay [130]. Originally identified as far upstream element 2 (FBP2), which derives FBP from far upstream elements of c-Myc (FUSE), KSRP was first described as a transcription factor and positive regulator of c-Myc in undifferentiated cells with an affinity to both DNA and RNA [131,132,133]. Two other members of the FBP family, FBP1 and FBP3, are also known to regulate c-Myc through transcription and splicing [130].

Structurally, KSRP is segmented into three regions: the proline/glycine-rich N-terminal, central nucleic acid-binding domain, and glutamine-rich C-terminal [134]. Within the central region, four KH domains (KH1–4) enable the binding of single-stranded nucleic acids, with the preferences of KH1 and KH3 to G- and GU-rich, KH2 to AU-rich, and KH4 to GA-rich sequences within RNA targets [134,135,136]. The less complex N- and C-terminal domains contain multiple post-translational modification sites which facilitate protein–protein interactions [130,132,137]. A 13-amino acid N-terminal domain NLS is present despite nuclear and cytoplasmic localization in a cell-type-specific manner [138]. Regulation of KSRP translocation is coordinated by four copies of Y-rich repeats (DYTKAWEEYYKK) within the C-terminal domain that allow complex formation with TFIIH and thus facilitate KSRP-FUSE interaction [134,139]. While KSRP affects the expression of more than 100 genes at multiple levels, its most well-documented function is its ability to bind to AREs within the 3′UTR of target transcripts to promote exonuclease-mediated mRNA decay and miRNA maturation [134,135,137,140,141].

Expression of KSRP plays a context-dependent role in several types of cancer [130,134,142]. Cancers showing over-expression of KSRP include small cell lung cancer (SCLC), non-small cell lung cancer (NSCLC), esophageal squamous cell carcinoma (ESCC), CRC, Glioblastoma, and clear cell renal cell carcinoma (RCC) [143,144,145,146,147,148]. mRNA decay is promoted through KSRP by binding of 3′UTR ARE sequences in NSCLC via Spry4 [149]. Packaging of snoRNA-KSRP into granules for enhanced translation contributes to PDAC formation [150]. KSRP also interacts with Neat1 lncRNA in STS [151]. The roles of KSRP in tumorigenesis extend beyond these mechanisms and cancer types.

KSRP acts on the Wnt signaling pathway in direct and indirect ways. Following confirmation of the 3′UTR-ARE specificity of KSRP by Gherzi et al. in 2004, the authors hypothesized its binding to *CTNNB1* mRNA to promote the recruitment of decay machinery [141]. KSRP was confirmed by Bikkavilli and Malbon in 2010 to directly interact with and destabilize *CTNNB1* mRNA (Figure 4). They also found that a protein–protein interaction occurs between Disheveled3 (DVL3) and KSRP, dependent on KSRP-CTNNB1 binding, which downregulates β-catenin expression in CRC cells [30].

The modulation of canonical Wnt signaling by KSRP-regulated miRNA is widely reported and further underscores its tumor suppressive role [152]. It has been demonstrated that KSRP controls the regulation of 28 different miRNAs [142,153]. Within Wnt signaling, KSRP regulates miR-1, miR-16, miR-21, miR140, miR148a, miR-192, miR-196a, miR-199a, miR-206, miR-221, miR-361, and miR-941, which in turn affects the expression of Wnt3a, GSK3 β, DKK2, TCF4, Wnt1, FZD9, possibly LRP5/6, FZD7, DKK2, Wnt10a, and Wnt16, respectively [154,155,156,157,158,159,160,161,162,163,164,165]. The maturation of Let-7 miRNA, of the Lin28/Let-7 axis, is also mediated by KSRP and can indirectly downregulate β-catenin expression, possibly a result of competitive binding between Lin28 and KSRP to Let-7 [142,166,167]. The destabilization of *PITX2* mRNA by KSRP is another less-understood indirect regulation KSRP has on Wnt signaling [168,169].

KSRP plays a uniquely dichotomous role in Wnt signaling downregulation. Through both the targeting of *CTNNB1* mRNA and the regulation of a plethora of miRNAs, KSRP has the potential to be a more appreciated contributor to canonical Wnt regulation in many cancers.

### 2.6. FMRP

Fragile X retardation protein (FMRP), encoded by *FMR1*, is an extensively studied, critical modulator of RNA stability and translation during neuronal development [170,171,172,173]. An aspect of almost every fragile X syndrome (FXS) case, the most common form of intellectual disability, is an expansion of 30 CGG repeats in the 5′UTR of *FMR1,* resulting in transcriptional deactivation and loss of FMRP [174]. Since the discovery of FMRP, extensive research has followed into its use as a therapeutic target in neurological disorders, and recently in cancers [175,176].

FMRP contains two central KH domains (KH1 and KH2) as well as a C-terminal RGG box [174,177,178]. At its extreme N-terminal, FMRP contains two tandem Agenet (Tudor-like) domains, which recognize methylated arginine or lysine to stabilize protein–protein and some protein–RNA interactions [179,180,181,182]. An NLS is followed just after its Agenet domains, as well as an NES just after its KH2, together allowing for shuttling between the nucleus and cytoplasm [183]. Between the NLS and KH1 domains resides a non-canonical KH0 domain, named for its KH-like folding and lack of a classical GXXG motif, which facilitates protein–protein interactions [182,184]. FMRP has also recently been established as a reader of the m^6^A RNA modification by recognition within the KH0 domain, with R138Q suppressing this interaction, and is required for nuclear export [174,185,186]. Furthermore, FMRP can regulate the RNA stability of m^6^A-modified targets in a YTHDF2-dependent manner [187].

While normally involved in translational repression in synapses by affecting ribosomal translocation [173,188], roles for FMRP in cancer have additionally been found. Overexpression of FMRP in mouse models using pancreas, colon, breast, and melanoma tumors has been shown to repress immune response [189]. Context-dependent aberrant expression of FMRP plays roles in a multitude of cancers, including CRC, clear cell renal cell carcinoma (ccRCC), melanoma, breast, prostate, esophageal, and others [190,191].

Underscoring its roles in neuronal development and cancers, FMRP acts directly and indirectly on canonical Wnt signaling. FMRP has been found to bind to the 3′UTR of *GSK3β* mRNA and repress its translation in neural progenitor cells [31]. In agreement with these results, FMRP binds directly to and stabilizes *CTNNB1* and *WNT5B* mRNAs, activating downstream signaling in glioblastoma (GBM) (Figure 4) [32]. FMRP also indirectly enhances β-catenin-targeted transcription through stabilization of DDX5 (p68) by blocking its ubiquitination in breast cancer [190,192]. Another recent study demonstrates that FMRP indirectly interacts with FZD3 protein through Celsr3 in neurons and is disrupted by WNT5a [193]. *APC* mRNA has been shown to colocalize with FMRP in axons [193,194], but direct binding has yet to be observed. Using RIP-seq in the cortical lysates of E14.5 aged mice, CTNNB1 and TCF4 were found to be targets of FMRP; however, other studies have yet to confirm *TCF4* mRNA as a direct binding partner of FMRP in humans [195]. Overlap between *FMRP* mRNA targets with TCF4 transcriptional targets suggests the possibility of an FMRP-mediated feed-forward loop [196,197]. In vascular smooth muscle cells, FMRP recruits β-catenin to repress translation of target mRNA [198]. Loss of *dfmr1* in *Drosophila* is associated with enhancement of Wnt signaling [199].

FMRP has been identified as an effector of the Wnt cascade through a variety of mechanisms. Given the multiple direct and indirect implications of FMRP on Wnt, these studies elucidate the emerging significance FMRP has on Wnt signaling components and highlight it as a potential clinical target. Due to the enhanced risk of cancer amongst Fragile X patients, a greater understanding of FMRP Wnt targets could prove useful in therapeutic development for these patients, as well as cancer patients.

### 2.7. Quaking

Quaking (QKI) proteins, members of the signal transduction and activation of RNA (STAR) class, as well as the hnRNP K homologous domain family (KH), find their roles in a variety of functions, including RNA stability and RNA splicing in multiple tissues [200]. The first reports of QKI describe the characteristic shaking or “Quaking” phenotype resulting from demyelination of the central nervous system in a spontaneous recessive mutant in mice [201].

The structure of QKI varies amongst its isoforms at their C-termini but remains constant upstream [202]. Within the QKI STAR region, a single, extended KH domain is flanked upstream by QUA1, a domain necessary for homodimerization and RNA binding, and downstream by QUA2, which works in conjunction with the KH to efficiently bind to target transcripts [200,203,204,205,206]. The N-terminal region contains proline-rich clusters as well as two RG boxes and is upstream of the STAR region. Downstream of the STAR region, the variable C-terminal region is separated by proline- and tyrosine-rich clusters as well as seven Src homology 3 (SH3) binding sites [202]. The pluripotent QKI locus has four protein isoforms named for the kilobase length of their transcripts: QKI-5, QKI-6, QKI-7, and the less-understood QKI-7b [202,207,208]. Each isoform is multifaceted, with QKI-5, QKI-6, and QKI-7 playing unique roles in cellular differentiation and signaling in multiple tissue types, often having overlapping spatiotemporal expression [200,202,207]. Notably, QKI-5 contains a nuclear localization signal within its C-terminal region. By recognizing cis quaking regulatory elements (QRE), QKI isoforms are able to regulate expression and splicing pattern of their respective target transcripts [209]. The putative QRE sequence, NACUAAY-N1–20-UAAY, has been identified in nearly 1430 mRNA targets [210]. Context-dependent homo- and hetero-dimerization is required for the binding of QRE sequences [207].

Growing evidence point to QKI proteins as regulators of Wnt signaling. Prior to its association with the Wnt signaling pathway, QKI had a well-defined role in Src-family protein kinase (Src-PKT)-dependent tyrosine phosphorylation-regulated RNA binding, displaying the possibility for a role in other signaling controlled processes [211,212]. QKI-7 was found to promote polyadenylation and efficient translation of target transcripts, underscoring the multifaceted functions of the QKI isoforms [213]. In contrast, QKI-5 was found to repress β-catenin translation as well as promote its degradation, inhibiting pro-metastatic phenotype through binding of its 3′ UTR in lung cancer [33]. The earliest report of QKI affecting components of the Wnt pathway comes upon the identification of the putative QRE sequence. QKI-5 and QKI-6 are negative regulators of β-catenin through binding of two QRE in its 3′ UTR, repressing its translation in CRC [34]. QKI was confirmed to regulate β-catenin translation as opposed to mRNA stability, such that its expression on the intramembrane would not be disruptive to endothelial barrier function [214]. A study investigating carcinogenesis in ulcerative colitis mouse models demonstrated that reduction in inflammation led to epigenetic upregulation of QKI alongside subsequent Wnt component downregulation, consistent with previous findings [62]. The regulatory relationship between QKI and Wnt signaling has also been shown in liver and renal cancers [215,216]. Recently, QKI has been shown to be an inhibitor of osteogenic differentiation in bone marrow stem cells via the downregulation of multiple key canonical and non-canonical Wnt pathway proteins [217]. Another recent study has shown a possibly indirect regulation of Wnt signaling and metastasis through QKI-mediated protection of *SLC7A7* mRNA, encoding a protein involved in arginine metabolism, and is posited to be through N7-methylguanosine (m7G) recognition (Figure 4) [218].

The role QKI has in Wnt signaling remains underexplored. Although, QKI is rapidly becoming a better-defined regulator of the Wnt cascade, studies elaborating on the spatiotemporal and isoform-specific roles of QKI in Wnt signaling are rare. Further research could be fruitful in the identification of novel mechanisms surrounding many aberrant Wnt-related cancers.

### 2.8. MEX3A

The dual-functional human muscle excess 3A (MEX3A) influences cell cycle progression, immunity, mRNA expression dynamics, and tumorigenesis through its RNA-binding activity, but also acts as a ubiquitin ligase [219,220,221,222]. The Mex3 family was first identified when mutations in the MEX-3 gene led to excessive muscle formation in the blastomeres of *C. elegans*, and was expanded to include four proteins (MEX3A-D) [223,224]. Its role has expanded into a viable prognostic disease marker and intriguing therapeutic target in cancer [225,226,227,228].

The four members of the Mex3 family contain two central KH domains to facilitate their RNA-binding activity [224,229]. Each also contains a C-terminal C3HC4-type RING finger domain to catalyze the transfer of ubiquitin to protein targets, including p53 [222,224,230]. All four contain an N-terminal NES sequence, while only MEX3B and C contain an NLS [224]. MEX3A nuclear shuttling is achieved in a CRM1-dependent manner [224,231]. Primarily through 3′UTR binding, MEX3A modulates the stability of target transcripts, depending on cellular context [229,232,233,234]. Predominantly through the effects it has on proliferation and stemness, MEX3A upregulation is associated with a variety of cancers, including CRC, ovarian, breast, ccRCC, and osteosarcoma [36,221,222,228,229].

MEX3A has various direct and indirect roles within canonical Wnt signaling, primarily through the binding of target mRNAs. Expression of MEX3A is associated with LGR5-positive cells within the mammalian intestinal crypt [235,236]. A recent study not only confirmed these previous observations but identified direct MEX3A binding to the 3′UTR of LGR5, stabilizing its expression, enhancing Wnt signaling and promoting intestinal crypt homeostasis [35]. Furthermore, this study also revealed clock gene BMAL1 to be a MEX3A transcription factor, expanding its role as a regulator of other components of canonical Wnt signaling [35,237].

Other studies show that expression of MEX3A correlates to upregulation of Wnt signaling components in several cancers including CRC, HCC, endometrial, and breast [36,228,235,238,239,240]. In breast cancer, MEX3A was shown to bind the 3′UTR of *DKK1* mRNA and repress its translation via Dcp-containing P-body dependent decay [36]. Another direct role for MEX3A includes its protein–protein interaction with DVL3 which stabilizes Wnt/β-catenin signaling and promotes tumorigenesis in endometrial carcinoma [239]. MEX3A has also been shown to bind to the 3′UTR of Wnt antagonist KLF4, suppressing its expression in CRC [238].

Overall, expression of MEX3A has been shown to have a strong positive correlation with the activation of canonical Wnt. This close association makes it an intriguing target in the development of treatments for highly proliferative, Wnt-driven cancers. Currently, no specific inhibitors of MEX3A’s RNA-binding function are available, highlighting the need for continued research and drug development in this area.

## 3. Discussion

This review highlights the increasing role of RBPs as modulators of canonical Wnt/β-catenin signaling and its impact on tumorigenesis. The canonical Wnt pathway has been well characterized, and it is now clear that post-transcriptional regulation by RBPs provides an indispensable layer of regulation. By influencing mRNA stability, translation, localization, and turnover, RBPs generate feed forward and feedback circuits that regulate the Wnt cascade in a context-specific manner.

Among these, IGF2BP1 stands out as a central regulatory hub. As a direct transcriptional target of β-catenin/TCF, IGF2BP1 integrates canonical pathway activation with post-transcriptional stabilization of numerous Wnt-related transcripts. By stabilizing target mRNA, IGF2BP1 amplifies pathway activity and supports oncogenic growth. Other RBPs also contribute to shaping Wnt pathway output. QKI isoforms generally suppress β-catenin by binding to its 3′UTR, while HuR enhances Wnt activity by stabilizing *LRP6* and *CTNNB1*. KSRP, in contrast, destabilizes Wnt-related transcripts and promotes the maturation of microRNAs that collectively downregulate the pathway. Members of the RBM family such as RBM3 and RBM47 display context-specific roles, acting as tumor suppressors in some settings and as oncogenic enhancers of Wnt in others. FMRP interacts with both *GSK3β* and *CTNNB1*, MSI1 represses *APC* and enhances Wnt signaling in epithelial progenitors, while MEX3A stabilizes *LGR5* and represses *DKK1*, thereby promoting stemness and tumor progression. These diverse examples reveal that Wnt signaling is not dictated solely by β-catenin abundance or transcriptional activation, but aided by a complex network of post-transcriptional regulators that determine which transcripts persist, where they localize, and how effectively they are translated.

Multiple Wnt/β-catenin inhibitors are currently under clinical/preclinical trials, each with distinct mechanisms. An FDA-approved antiparasitic drug Niclosamide, suppresses tumor growth and metastasis by targeting FZD1, DVL2, and LRP6 with minimal animal toxicity. Wnt inhibition can also increase immune infiltration in tumors that leads to enhanced antitumor immunity. Natural compounds such as CPG049090 and PKF222-815 block β-catenin/TCF interactions, while iCRT molecules disrupt β-catenin responsive transcription, though in vivo evidence remains limited. TNIK inhibition (NCB-0846) reduces Wnt-driven tumorigenesis, and the cyclic AMP-response element-binding protein (CBP)- β-catenin antagonist ICG-001, and its clinical derivative PRI-724, demonstrates anticancer activity in preclinical and early clinical trials. Overall, diverse preclinical and clinical inhibitors are emerging, offering therapeutic potential specifically designed to cure cancer [11].

RBPs represent a promising group of novel therapeutic targets affecting Wnt signaling. Because many are oncofetal proteins with low expression in adult tissues but high re-expression in tumors, they may provide therapeutic windows with reduced toxicity. Small molecules that disrupt RBP–RNA interactions, such as those developed against IGF2BP1 or HuR, could inhibit Wnt-driven oncogenic programs without fully shutting down upstream signaling. Nevertheless, several limitations remain with the activity of RBPs, as it is profoundly context-dependent, varying across tissues, tumor types, and environmental stresses.

Combining RBP inhibitors with Wnt pathway inhibitors offers a potent strategy to combat cancers by targeting multiple regulatory layers. While Wnt inhibitors, such as those disrupting β-catenin/TCF interactions, blocking coactivators like CBP, or inhibiting kinases like TNIK, suppress tumor growth and enhance immune infiltration by disrupting key signaling pathways, RBP inhibitors interfere with post-transcriptional regulation, affecting RNA stability and the translation of Wnt components and related oncogenes. This dual targeting can improve therapeutic efficacy and reduce resistance arising from pathway redundancy. However, challenges include the limited availability of selective RBP inhibitors, potential off-target effects due to RBPs’ broad roles within cells, and the need to balance Wnt inhibition to avoid impairing normal stem cell function. Managing inhibitor use based on cancer type and stage is crucial. Together, these approaches build on the progress of Wnt inhibitors, aiming to enhance antitumor effects while modulating the tumor microenvironment more effectively.

## Figures and Tables

**Table 1:** Summary of canonical Wnt components whose mRNAs are directly targeted by RBPs. ‘S’ refers to target transcript stability, and ‘T’ refers to translatability of the target transcript. Arrows pointing up refer to upregulation and arrows pointing down refer to downregulation.

RBP	Targets	Regulation	Target Region	Model Cell Line	References
*IGF2BP1*	*βTRCP*	↑ S	CDS	HCT116	[20]
	*GSK3β*	↑ S	undetermined	C33AA	[21]
*ELAVL1*	*LRP6*	↑ S, ↑ T	3′UTR	IEC-6	[22]
	*CTNNB1*	↑ S, ↑ T	undetermined	BMSCs	[23]
*MSI1*	*APC*	↓ T	3′UTR	NIH3T3	[24]
				HCT116, HEK293	[25]
	*CTNNB1*	↓ T	3′UTR	HCT116, HEK293, Mouse Epithelium (in vitro)	[25]
*MSI2*	*LEF1*	↓ S	undetermined	K562	[26]
*RBM3*	*CTNNB1*	↓ S	3′UTR	PC-3	[27]
*RBM47*	*DKK1*	↑ S	3′UTR	231-BrM2	[28]
	*AXIN1*	↑ S	3′UTR	H1299 and A549	[29]
*KSRP*	*CTNNB1*	↓ S	3′UTR	F9	[30]
*FMRP*	*GSK3β*	↓ T	3′UTR	aNPCs (mouse-derived)	[31]
	*CTNNB1*	↑ S	undetermined	GSCs (patient-derived), T98G	[32]
*QKI 5/6/7*	*CTNNB1*	↓ T	3′UTR	HT29	[33,34]
*MEX3A*	*LGR5*	↑ S	CDS, 3′UTR	mIECs	[35]
	*DKK1*	↓ S, ↓ T	3′UTR	BT549, MDA-MB-468, SUM159PT	[36]

## Data Availability

No new data were created or analyzed in this study. Data sharing is not applicable to this article.
